# The effect of acupuncture on the quality of life of patients recovering from COVID-19: A systematic review protocol

**DOI:** 10.1097/MD.0000000000020780

**Published:** 2020-07-24

**Authors:** Dengpeng Wen, Liu Wu, Yuting Dong, Ju Huang, Kuiyu Ren, Jianzhen Jiang, Shunxin Dai, Wei Zhao, Xinwei Xu, Dezhong Peng

**Affiliations:** a College of Acupuncture and Tuina, Chengdu University of Traditional Chinese Medicine; b Department of Tuina, Hospital of Chengdu University of Traditional Chinese Medicine, Chengdu, Sichuan province, P.R. China.

**Keywords:** acupuncture, COVID-19, systematic review

## Abstract

**Background:**

Assessing the effectiveness and safety of acupuncture therapy for treating patients with COVID-19 is the main purpose of this systematic review protocol.

**Methods:**

The following electronic databases will be searched from inception to May 2020: Cochrane Central Register of Controlled Trials, PubMed, Web of Science, EMBASE, China National Knowledge Infrastructure, Traditional Chinese Medicine, Chinese Biomedical Literature Database, Wan-Fang Database, and Chinese Scientific Journal Database. All published randomized controlled trials in English or Chinese related to acupuncture for COVID-19 will be included. Primary outcomes are timing of the disappearance of the main symptoms (including fever, asthenia, cough disappearance rate, and temperature recovery time), and serum cytokine levels. Secondary outcomes are timing of the disappearance of accompanying symptoms (such as myalgia, expectoration, stuffiness, runny nose, pharyngalgia, anhelation, chest distress, dyspnea, crackles, headache, nausea, vomiting, anorexia, diarrhea), negative COVID-19 results rates on two consecutive occasions (not on the same day), CT image improvement, average hospitalization time, occurrence rate of common type to severe form, clinical cure rate, and mortality.

**Results:**

The results will provide a high-quality synthesis of current evidence for researchers in this subject area.

**Conclusion:**

The conclusion of our study will provide an evidence to judge whether acupuncture is an effective intervention for patients suffered from COVID-19.

**Ethics and dissemination:**

Formal ethical approval is not necessary as the data cannot be individualized. The results of this protocol will be disseminated in a peer-reviewed journal or presented at relevant conferences.

**PROSPERO registration number:**

CRD42020183736.

## 1 Introduction

Coronoviruses have been reported as causes of mild and moderate respiratory infections for >50 years.^[1]^ Even though this group of viruses have been isolated from many different animals, bats are accepted major natural reservoir of coronaviruses.^[2,3]^ Four human coronavirus, 229E, HKU1, NL63, and OC43 are known as causes of common cold in humans.^[1]^ However, recently detected coronaviruses, SARS CoV (2002) and MERS-CoV (2012) completely altered all known approaches about this virus group because these viruses caused severe acute respiratory infections and nosocomial outbreaks. In the end of 2019, a novel coronavirus, now known as SARS-CoV-2 (2019), suddenly emerged in Wuhan, China. The World Health Organization declared that the epidemic is a public health emergency of international concern on January 31, 2020. As of April 16, 2020, the emerging coronavirus infection, COVID-19, has been spreading worldwide, causing >2 million cases and >137 thousand of death. The symptoms of COVID-19 infection appear after an incubation period of approximately 5.2 days.^[4]^ The period from the onset of COVID-19 symptoms to death ranged from 6 to 41 days with a median of 14 days.^[5]^ This period is dependent on the age of the patient and status of the patient's immune system. It was shorter among patients >70 years’ old compared with those under the age of 70.^[5]^ The most common symptoms at onset of COVID-19 illness are fever, cough, and fatigue, whereas other symptoms include sputum production, headache, hemoptysis, diarrhea, dyspnea, and lymphopenia.^[5–8]^ Clinical features revealed by a chest CT scan presented as pneumonia; however, there were abnormal features such as RNAaemia, acute respiratory distress syndrome, acute cardiac injury, and incidence of grand-glass opacities that led to death.^[6]^ In some cases, the multiple peripheral ground-glass opacities were observed in subpleural regions of both lungs^[9]^ that likely induced both systemic and localized immune response that led to increased inflammation. Regrettably, treatment of some cases with interferon inhalation showed no clinical effect and instead appeared to worsen the condition by progressing pulmonary opacities^[9]^: COVID-19 has been reported form all aged patients. Severity of infection could be varied from asymptomatic infection to critical disease. Clinical severity of COVID-19 was defined in 5 groups as asymptomatic, mild, moderate, severe, and critical. Diagnostic criteria of these groups were as follows^[10,11]^: asymptomatic infection, that is, without any clinical sign of symptoms with positive SARSCoV-2 PCR test; Mild, that is, symptoms of acute upper respiratory tract infection, including fever, fatigue, myalgia, cough, sore throat, runny nose, and sneezing without pneumonia; moderate, that is, with pneumonia, frequent fever and cough (some may have wheezing, but no obvious hypoxemia such as shortness of breath); Severe, that is, rapid progression around 1 week, dyspnea, with central cyanosis, oxygen saturation <92%, with other manifestations of hypoxemia; Critical, that is, patients with acute respiratory distress syndrome or respiratory failure, shock, multiple organ dysfunction. This clinical classification is also important because it gives some clues about prognosis and mortality of COVID-19. Most COVID-19 cases (81%) were classified as mild or moderate disease in adults, and in children most cases were mild.^[10,12,13]^ In critical cases mortality could be as higher as 50% in adults.^[10,12]^ Acupuncture is an effective,^[14–16]^ inexpensive,^[17]^ and safe treatment, with no side effects or infections have been reported^[18]^; it has been widely used in China for thousands of years and has been accepted worldwide. Acupuncture has a long history of use in China, Japan, and Korea. Contemporary acupuncture practice is commonly undertaken as part of the medical hospital system in modern China (Robinson2012); Traditional Chinese Medicine theory describes a state of health maintained by a balance of energy within the body. Acupuncture involves insertion of fine needles into different parts of the body to correct the imbalance of energy within the body.^[19]^

Currently, there is a lack of evidence-based medical evidence for the treatment of COVID-19 in convalescent patients. It is urgent for improvement of temper management, depression, and quality of life in convalescent patients. It is necessary to make a systematic review to provide a convincing conclusion whether acupuncture is an appropriate method to treat COVID-19.

## 2 Methods and analysis

### 2.1 Study registration

This systematic review protocol was registered with PROSPERO 2020 (registration number: CRD42020183736). And the protocol report is in the base of the Preferred Reporting Items for Systematic Reviews and Meta-Analyses Protocols (PRISMA-P) declaration guidelines.^[20]^ The review will be performed in line with the PRISMA declaration guidelines.^[21]^

### 2.2 Inclusion criteria for study selection

#### 2.2.1 Type of study

RCTs of acupuncture therapy for COVID-19 without restrictions on publication status will be eligible for inclusion.

#### 2.2.2 Type of participant

Participants who were 16 years or older with COVID-19 will be included in spite of the sex, race, education, or economic status.

#### 2.2.3 Type of intervention

Acupuncture therapy includes manual acupuncture, body acupuncture, electroacupuncture, plum blossom needle, warm needling, and fire needling. Other methods include transcutaneous electrical nerve stimulation, laser acupuncture, dry needling, cupping, and moxibustion will be excluded.

Comparison interventions, including sham acupuncture (including sham acupuncture at selected acupoints, sham acupuncture at non-acupoints, pseudo-acupuncture interventions, needling at inappropriate acupoints and nonpenetrating sham acupuncture), placebo, usual care, medication, no treatment, and other conventional therapies, will be included.^[22]^ In addition, the review of trials evaluating acupuncture combined with another treatment compared with other typical treatments alone will be included.

### 2.3 Searching other resources

The reference lists of potentially missing eligible studies will be scanned ant the relevant conference proceedings will be scanned as well.

#### 2.3.1 Search strategy

The search strategy for PubMed is shown in Table 1. The following search keywords will be used: COVID-19 (eg, “Corona Virus Disease 2019” or “Corona Virus ”;acupuncture (eg, “acupuncture” or “acupuncture therapy” or “body acupuncture” or “manual acupuncture” or “electroacupuncture or “fire needling” or “plum blossom needling”; randomized controlled trial (eg, “randomized controlled trial” or “controlled clinical trial” or “random allocation” or “randomized” or “randomly” or “double-blind method” or “single-blind method” or “clinical trial”. The equivalent search keywords will be used in the Chinese databases.

**Table 1:**
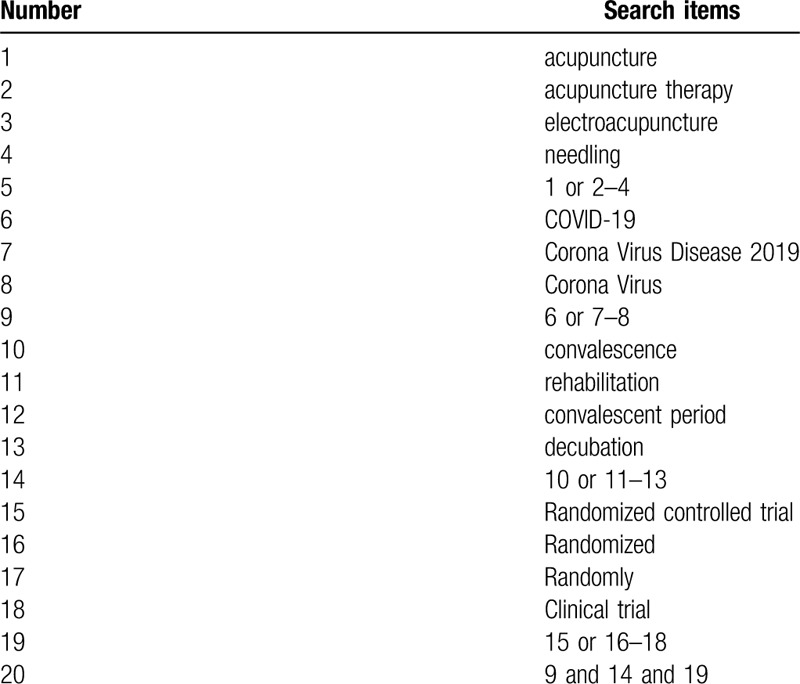
Search strategy for the PubMed database.

### 2.4 Data collection and analysis

#### 2.4.1 Selection of studies

**Figure 1:**
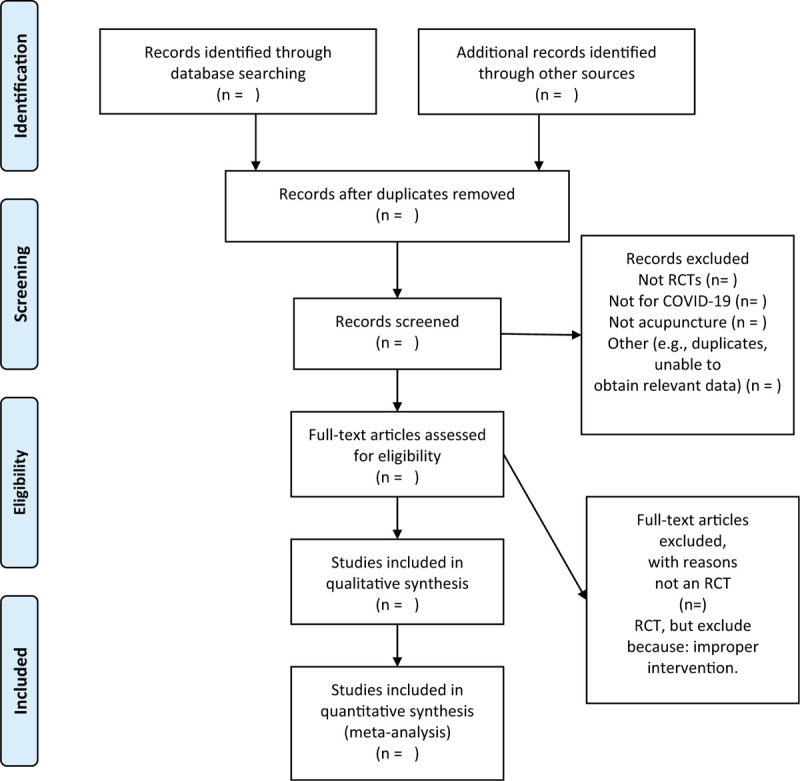
The flow diagram.

The titles and abstracts of all searched studies will be reviewed and screened independently by 2 reviewers, aiming at identifying eligible trials and eliminating duplicated or irrelevant studies in line with the criteria; the full text of all possibly eligible studies will obtained if necessary. A discussion with the third reviewer is planned to solve the disagreements. A PRISMA flow diagram will be used to show the study selection process (Fig. 1).

### 2.5 Data extraction and management

Before the data extraction, a standard data extraction form with following information will be created: year of publication, general information, country, participant characteristics, inclusion and exclusion criteria, sample size, methods, randomization, blinding methods, type of acupuncture interventions, control, outcome measures, results, adverse reactions, conflicts of interest, ethical approval, and other information. And these data will be extracted by 2 independent reviewers. A third reviewer will be set to be discussed with and judge the disagreements during the course. Contacting the authors for further information will be the solution for the insufficient data in the publications. Review Manager software (RevMan V.5.2.1) will conduct the analysis and synthesis after transferring all the data into the software.

### 2.6 Assessment of risk of bias in included studies

Two reviewers will independently evaluate the risk of bias in all included studies with the Cochrane Collaboration's tool assessment method and complete the Standards for Reporting Interventions in Clinical Trials of Acupuncture checklist for the studies included.^[23]^ The following domains will be evaluated: selection bias, performance bias, attrition bias, detection bias, reporting bias and other sources of bias. The assessments will then be divided into three levels: low risk, high risk, and unclear. Unclear or insufficient items will be obtained by contacting the corresponding author for further information. The third reviewer will be set to solve the disagreements.

### 2.7 Measures of treatment effect

Dichotomous data will be presented as risk ratio and 95% confidence intervals (CI), whereas continuous outcomes will be showed as standard mean difference 95 % CI.

### 2.8 Unit of analysis issues

The individual participant will the analytical unit.

### 2.9 Management of missing data

Contacting the corresponding authors of the included studies will be the solution to obtain the missing or insufficient data of the primary results including sending emails or making a call. If missing data is not available, an intent-to-treat analysis will be performed as much as possible (the analysis should include data from all participants in the initially randomly assigned group) and a sensitivity analysis will be performed to determine if the results are inconsistent.

### 2.10 Assessment of heterogeneity

*I*^2^ test will be used to quantify inconsistency and standard *χ*^2^ test will be used to detect statistical heterogeneity. Studies will be considered to have homogeneity if the *P* value exceeds .1 and the *I*^2^ value is <50%, and the fixed-effects model will be used, whereas studies will be considered to have significant statistic heterogeneity if the *P* value is <.1 or the *I*^2^ value exceeds 50%, and subgroup analysis will be used to explore the possible cause. And the random-effects model will be applied if the heterogeneity is still important.

### 2.11 Assessment of reporting biases

Funnel plots will be used to assess the reporting biases if >10 studies are included.

### 2.12 Data synthesis

Review Manager software (RevMan V.52) will be used to perform the data synthesis, if it is possible to carry out a meta-analysis. If no substantial statistical heterogeneity is detected, the data synthesis will be processed with the fixed-effects model, and if substantial statistical heterogeneity is detected, the data synthesis will be performed with the random-effects model. Possible reasons will be searched from a clinical and methodological perspective if different studies exist significant heterogeneity, and descriptive analysis or subgroup analysis will be provided. If there is no substantial heterogeneity between 2 studies, descriptive analysis will be conducted.

#### 2.12.1 Subgroup analysis

Subgroup analysis will be conducted if the data are sufficient, according to the factors different outcomes and different control interventions.

#### 2.12.2 Sensitivity analysis

Sensitivity analyses will be performed to evaluate the impact of sample size, study design, methodological quality and the effect of missing data, and to verify the robustness of the review conclusions If possible. The analysis will be repeated after low-quality studies are excluded.

### 2.13 Grading the quality of evidence

The Grade of Recommendations Assessment, Development and Evaluation (GRADE) will be the tool to evaluate the quality of the evidence.^[24]^ Limitation of study design, inconsistency of results, indirectness, imprecision, and publication bias will be assessed. The assessments will be divided into 4 levels: very low, low, moderate, or high.

### 2.14 Ethics and dissemination

Formal ethical approval is not necessary as the data cannot be individualized. The results of this protocol will be disseminated in a peer-reviewed journal or presented at relevant conferences. The essential protocol amendments will be documented in the full review.

## 3 Discussion

This systematic review will assess the effectiveness of acupuncture for COVID-19. There are 4 sections in the review: identification, study inclusion, data extraction and data synthesis. This review will help the doctors to choose acupuncture as an alternative treatment for COVID-19 patients, and offer the patients more options to relieve their symptoms.

## Author contributions

DPW and LW mainly contributed to this manuscript and joint first authors. DZP obtained funding. KYR, JZJ and SXD drafted the protocol. WZ,JH,and XWX make the search strategy and it will be conducted by them. KYR and JZJ will obtain copies of the studies and WZ and YTD will screen the studies to be included. Data extraction from the studies will be done by SXD and JZJ. KYR and JZJ will put the data into RevMan. Analyses will be conducted by XWX and WZ and KYR will interpret the them. DPW and LW will draft the final review and XWX and DZP will update the review. DZP will act as an arbiter in the study selection stage. All authors have read and approved the final manuscript.
